# University Day—1906

**Published:** 1906-02

**Authors:** 


### University Day — 1906.

THE University of Buffalo celebrates each year the twenty-second of February—which, on this account, has come to be

The University of Buffalo celebrates each year the twenty-second of February—which, on this account, has come to be known as University Day. The medical department instituted the custom February 22, 1899, but since that time all the departments have joined in the celebration which makes the name adopted most appropriate.

This year the celebration was not only patriotic because of the anniversary of the birth of the Father of his Country, but enthusiastic because it spoke in every detail for an enlarged university, a university of letters as well as law, of philosophy as well as physic, of arts as well as apothecaries, of doctors of divinity as well as doctors of dentistry. The students of the university to the number of 500 marched from the High Street buildings to the Teck Theater and there formed in two lines between which a procession, led by Vice-Chancellor Norton and Mayor Adam, consisting of city officials, military men, and citizens in general marshalled by Major General Welch, entered the building to music by the sixty-fifth regiment band.

The vast auditorium was soon filled, the boxes being occupied by prominent women who added their graceful presence to the occasion. At the appointed hour, 11 o'clock, Vice-Chancellor Norton called the assemblage to order, and Bishop Walker offered prayer. Then Mayor Adam made an excellent address, the burden of which was a plea for greater educational advantages for the young, and especially for the establishment of a college of arts as a part of the University of Buffalo. The Vice-Chancellor, who next took the stage, was received with a royal greeting by the audience, collegians, women, military men, civilians and city officials all joining to do honor to the man who had worked so hard, and accomplished so much, during the past year to bring about the establishment of an enlarged university. Mr. Norton's speech was one of the best of the many he has made on the subject, and when he had finished he again received the generous plaudits of the audience.

The university glee and mandolin clubs did their parts to make the occasion interesting, which all agreed was the best observance of University Day yet witnessed. With the singing of America the exercises closed.

---

Dr. DeWitt G. Wilcox, president of the Homeopathic Medical Society of the State of New York asks a few interesting questions of the editor of the Buffalo Commercial, in a recent issue of that venerable and esteemed newspaper:

"Why is there anything irregular in a physician's being interested in the sale of patent medicines?" asks the Commercial in a short editorial a few days ago. Why is there anything irregular in a decent editor's being interested in a blackmailing sheet or yellow journalism? Why is there anything irregular in an honest banker's being interested in a "get-rich-quick" concern or a "bucketshop?" Why is there anything irregular in a reputable lawyer's being interested in a "divorce mill?" Why is there any thing irregular in a trusted merchant's being interested in the sale of smuggled goods? Why? Because all of the above mentioned pursuits are either irregular, dishonest, fraudulent or criminal, created and pursued for the sole purpose of extorting or obtaining money from the ignorant, the unfortunate, the defenceless or the gullible. It seems scarcely credible that any intelligent person would seriously ask such a question in the light of present day facts—facts that are absolutely indisputable as to the fraud of ninety per cent. of patent medicines.

This seems to close the incident without further comment. Dr. Wilcox, in his recent presidential address at Albany, discussed many interesting problems which could be considered by all physicians, some of which are ably handled. In regard to his suggested method of uniting the several so-called schools of medicine, it does not seem to us that he is at his best ; at least he has not presented a plan that appears practicable. However, we cannot discuss the matter in extenso at this time.

---

The United States Civil Service Commission announces an examination at the usual places, to secure eligibles from which to make certification to fill vacancies as they occur in the position of copyist in the Bureau of Pensions. The age limit is 20 to 35 years, the salary is $900 a year, and only male graduates of recognised medical schools may be examined.

